# Studying Motivation in ADHD: The Role of Internal Motives and the Relevance of Self Determination Theory

**DOI:** 10.1177/10870547211050948

**Published:** 2021-11-19

**Authors:** Sarah Morsink, Saskia Van der Oord, Inge Antrop, Marina Danckaerts, Anouk Scheres

**Affiliations:** 1 KU Leuven, Leuven, Belgium; 2 University of Amsterdam, The Netherlands; 3 Gent University, Belgium; 4 Radboud University, Nijmegen, The Netherlands

**Keywords:** ADHD, motivation, self determination theory, reinforcement, reward

## Abstract

**Objective:**

Motivation is what moves us to act, what engages us in goal-directed behavior. The Self Determination Theory (SDT) is a motivational framework conceptualizing motivation—or internal motives—as a continuum of motivation qualities fueled by satisfaction of the three basic psychological needs Autonomy, Relatedness, and Competence. ADHD has been associated with motivational alterations that contribute to academic difficulties. However, ADHD theories and research are mainly focused on the effects of reinforcement on behavior, with little attention for the broader definition of motivation, that is, internal motives. Therefore, the main objective here was to introduce the SDT as theoretical framework within which we can develop relevant research questions about motivation in the field of ADHD.

**Method:**

To this end, we (i) present the SDT as a comprehensive motivational framework, and (ii) describe current motivation-related ADHD theories and research.

**Results:**

Based on this, we suggest how SDT can be used as a guiding framework in generating relevant research questions that can help broaden our understanding of the role motivation plays in individuals with ADHD.

**Conclusion:**

We conclude that ADHD research on motivation would benefit from (i) including internal motives as potential key mediators in the relation between environmental factors and behavior/symptoms; (ii) studying potential negative effects of external reinforcers intrinsic motivation, affect, and well-being. Finally, we conclude that this framework carries value for further development of clinical interventions for those with ADHD.

## Introduction

**Figure 1.**
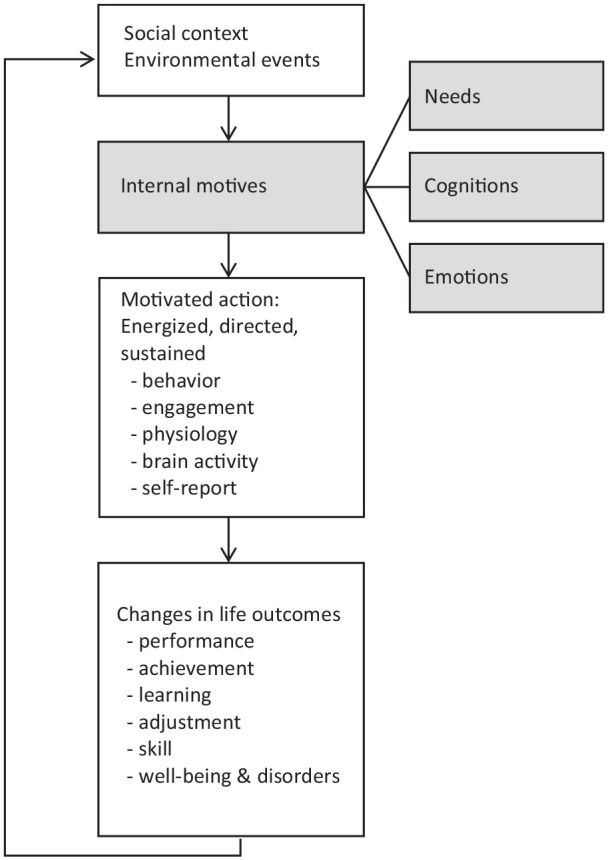
Framework to understand motivation. Based on Reeve (2014, Figure 1.2, p. 9; Figure 1.4, p. 16).

Motivation is a concept that is used to explain behavior, and it generally refers to that what moves us to act, what causes goal-directed behavior (Deci & Ryan, 2000, 2002; Reeve, 2014; Wigfield & Eccles, 2000). More specifically, motivation has been defined as *internal motives* that give behavior direction (i.e., behavior is aimed at an outcome or a goal), energy (i.e., behavior can vary in engagement intensity, or strength), and persistence (i.e., endurance of engagement over time) (Reeve, 2014). These internal motives (needs, cognitions, emotions) serve as mediators between external triggers on the one hand and behavior or performance on the other (see Figure 1). For example, a parent asks her child to do homework. In response, the child starts doing homework. The parental request does not *directly* result in the child’s engagement in homework, but rather indirectly: the effect of the parent’s request on the child’s performance depends on internal motives of the child; for example cognitions on the usefulness of homework, emotions related to homework completion, and the extent to which homework can attend to a child’s needs (Reeve, 2014) (Figure 1). Thus, external triggers such as parents’ requests, can provide supportive or frustrating conditions, which affect internal motives that drive behavior. Given that motives are internal, indirect manifestations of this latent concept are typically measured in order to infer the contributing role of these internal motives. Aspects of behavior, engagement, psychophysiology, brain activity, and self-reports are examples of indirect manifestations of these internal motives (Reeve, 2014).

Internal motives are not explicitly operationalized in the majority of ADHD-related motivational frameworks and research, while both theories and empirical work have suggested that motivation may be altered in individuals with ADHD (Luman et al., 2010; Plamondon & Martinussen, 2019; Smith & Langberg, 2018). Such motivational alterations are thought to play a role in ADHD, in addition to and in interaction with cognitive impairments (Barkley, 2015; Nigg et al., 2002), resulting in suboptimal functioning in everyday life, for example in the academic context (Biederman et al., 2004; Langberg et al., 2013). ADHD research so far is largely limited to the measurement of direct effects of external rewards and punishments on behavior and performance, hereby bypassing the mediational effect of these internal motives on the behavioral expression and performance. Therefore we believe that future ADHD theories and research on motivation can benefit from broader theoretical frameworks that explicitly include internal motives in the conceptualization and operationalization of motivation. The Self-Determination Theory (SDT) is such a candidate theory that could enrich motivation research in individuals with ADHD that way.

**Figure 2.**
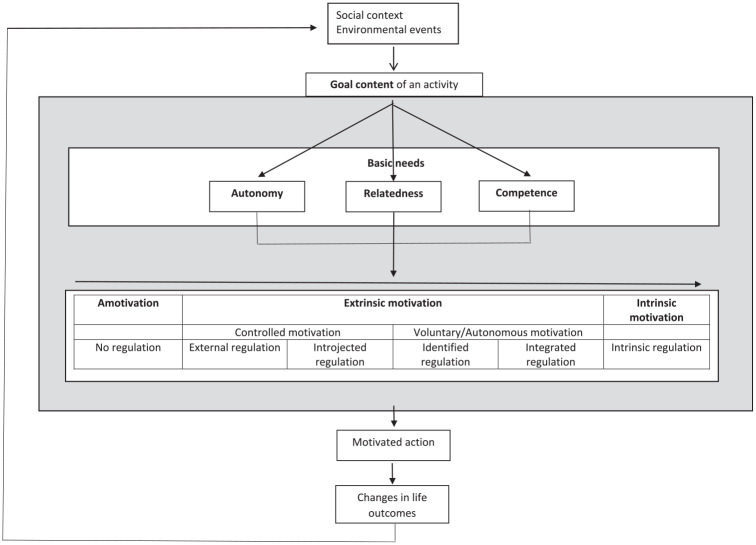
Schematic representation of the way SDT conceptualizes internal motives based on the model of Reeve (2014). The social context gives rise to (personal interpretation of) goals present in relation to the activity (see Goal Content Theory). These activity characteristics can support/undermine basic need (BN) satisfaction or frustration (see Basic Needs Theory) which leads to the internal growth tendency visible in increased motivation quality (see Organismic Integration Theory). These internal motives influence motivated action, and changes in life outcomes. Due to experience, one regulation style (see Causality Orientations Theory) can dominate inter-individually resulting in the differences in the pursuit/interpretation of activities/goals.

**Figure 3.**
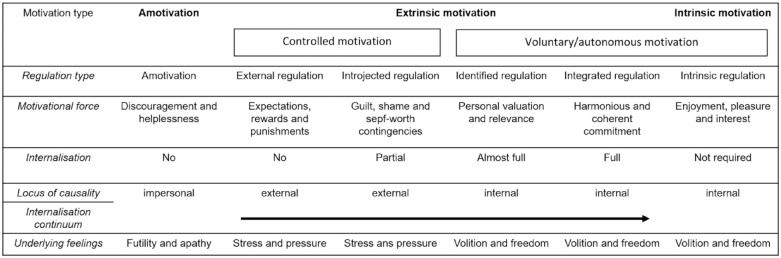
Schematic representation of the 6 types of motivation. Adapted from Vansteenkiste et al. (2010, p. 115).

This theory is one of the most influential motivational frameworks that defines motivation as a natural internal human tendency toward growth (Deci & Ryan, 1987). This tendency gives energy, direction, and persistence to behavior and thus refers to the broad conceptualization of internal motives or motivation (Reeve, 2014). The SDT argues that the human internal tendency toward growth expresses itself as autonomous choices to engage and take part in activities. In this theory, motivation is defined as a continuum of motivation qualities ranging from more controlled (i.e., activity engagement which originates from a sense of pressure) to more voluntary (i.e., engaging in activities because of personal enjoyment with or interest in the activity) motivation forms. This dimension of motivation qualities is fueled by the satisfaction or frustration of innate basic psychological needs: Competence, Autonomy, and Relatedness (Deci & Ryan, 1987). The more the environment is able to satisfy these needs, the more voluntary forms of motivation can flourish, and the higher levels of wellbeing individuals will experience. An example of the need for “relatedness” being satisfied is a child feeling that the parent is involved and interested in his/her wellbeing to an extent that feels good. On the other hand, the more these needs are frustrated, the harder it is to feel voluntarily motivated. An example of the need for “autonomy” being thwarted is a child feeling often that (s)he is forced to do things (s)he does not want to do. When needs are frustrated to a large extent—and the natural tendency toward growth is hampered—psychopathology might occur (Vansteenkiste, Lens, & Deci, 2006). To come back to the example of homework: if engagement in homework will result in feelings of competence or relatedness with others, the student will be more likely to experience voluntary motivation than when engagement in homework only involves the escape of a punishment (see Figure 2: summarized model of SDT; for a detailed explanation of the theory, see below; see Figure 3).

SDT-based research in ADHD is still very scarce. First, Rogers and Tannock (2018) found that in a non-clinical sample of primary school students, higher levels of ADHD symptoms were associated with lower satisfaction of all three basic psychological needs in the classroom, as measured with a self-report rating scale. An SDT-informed interpretation would be that such low need satisfaction might arise from need thwarting (Ryan et al., 2016). An example of thwarting of the need relatedness is a child feeling rejected by peers. Second, Morsink et al. (in preparation ) used an experimental design in participants with ADHD and typically developing peers. They found a positive effect of task instructions that included the support of the psychological basic needs in its language (as compared to undermining instructions) on self-reported motivation, task engagement, and subjectively experienced enjoyment during a working memory task. This effect was present in all participants. Third, Carlson et al. (2000) used and arithmetic task and examined the effects of reward and response cost in children with ADHD and controls not only on performance, but also on intrinsic motivation. This was measured by voluntary engagement in this task, after having completed the task under varying contingencies. Based on SDT, they hypothesized that intrinsic motivation for the task would decrease after inclusion of performance-contingent reinforcement (known as the undermining effect) with a potential differential effect on children with ADHD. The authors reasoned that this question is particularly relevant for children with ADHD “because they frequently show a lack of persistence in the face of effortful tasks” (p. 88). Contrary to their hypothesis, no undermining effects of reinforcement on intrinsic motivation were found, as demonstrated by the fact that the free choice to engage in the arithmetic task was the same whether this choice was preceded by a rewarded or unrewarded arithmetic task. Why Carlson and colleagues did not find an undermining effect of the performance-based and participation-based contingencies on intrinsic motivation is still an open question. Perhaps, and speculatively, because the contingencies (tokens) were accompanied by verbal positive feedback (in the article it is reported as “feedback/contingencies”), this may have canceled out any undermining effect of the tokens. Or perhaps it can be explained by the fact that the arithmetic task was not intrinsically motivating to participants to begin with and therefore there was not much intrinsic motivation to be undermined (floor effect). This may have hampered the ability to measure any undermining effect of external reinforcers on intrinsic motivation. Therefore, Carlson and Tamm (2000) conducted a follow-up study using the same design where task interest was manipulated by including both an uninteresting task and an interesting one. Unexpectedly, reward or response cost did not have detrimental effects on intrinsic motivation for the interesting task (nor for the uninteresting one) for both groups.

It is the aim of the current paper to present the SDT as a comprehensive framework that can enrich current ADHD theories and research. More specifically, we will (i) introduce the SDT in more detail, (ii) describe current motivation-related ADHD theories and research in more detail, and then (iii) outline how SDT can be used as a guiding framework in suggesting research questions that can help broaden our understanding of the role motivation plays in individuals with ADHD.

## SDT: The Five Mini-Theories

SDT is a macro theory of motivation that is built up from five dissociable mini-theories developed and integrated over time (Vansteenkiste et al., 2010). These mini-theories and their link to current ADHD research are explained extensively in the following paragraphs.

### Organismic Integration Theory

This mini theory distinguishes various qualities, or types of motivation ranging from a-motivation to intrinsic motivation, with extrinsic motivation in between. Within extrinsic motivation, various qualities can be distinguished, ranging from controlled to voluntary/autonomous (Figure 3). According to this mini theory, these motivation qualities relate to the perceived locus of causality (Deci & Ryan, 2000) (Figure 3). The left extreme concerns *A-motivation*, this is non-intentional behavior arising from (i) the idea that behavior is not linked with perceived contingency, (ii) feeling incapable to perform the behavior, or (iii) a lack of valuation of the activity (Deci & Ryan, 1985a). Here the locus of causality is only external. The right extreme is *Intrinsic motivation*, which comprises behaviors that are engaged in only out of feelings of joy and interest, originating from an internal causality (Deci & Ryan, 2000). Here, behaviors are seen as self-regulated (de Charms, 1968), and can be linked with feelings of volition and freedom (Vansteenkiste et al., 2010).

Between a-motivation and intrinsic motivation, four additional qualities are distinguished, based on the degree to which *the reasons* to perform the behavior are internalized, that is the degree to which the locus of control is perceived as internal (Deci & Ryan, 2000). Each of these motivation types is accompanied with specific internal motives, namely emotions and cognitions (de Charms, 1968) (Figures 2 and 3: schematic representation of motivation qualities). In other words, internal motives are part of each form of motivation, independent of the degree to which the reasons to perform behavior are internalized. *External regulation* refers to behavior that is performed solely under control of external reward or punishment—but still via internal motives (Deci & Ryan, 1980). For example, a student might do homework because his/her parents promised him/her a new video game, and the internal motive could be happy feelings associated with anticipating the video game. In this situation, behavior will only be maintained as long as that reinforcer is pending. Second, *Introjected regulation* occurs when the contingency is no longer pressured from the outside but from within the person, in an attempt to experience feelings of pride, and to avoid feelings of guilt and shame (Deci & Ryan, 1980). For example, the student might do homework because that is what students are supposed to do. External and introjected regulation together are also referred to as *controlled motivation*. Third, *Identified regulation* presents itself when the individual understands the value of the behavior (Deci & Ryan, 1980). The perceived locus of control is internal, and thus the behavior is accompanied with feelings of volition and freedom (Vansteenkiste et al., 2010). For example, the student finds the homework task relevant for his/her future academic career. Fourth, *Integrated regulation* implies integration of the meaning of the behavior with other aspects of the self (Deci, 1976). For example, the student sees him or herself as someone for whom applying effort is important. Identified and integrated regulation together are also referred to as *autonomous motivation*.

Thus, the internalization continuum ranges from external to integrated regulation and comprises behaviors that are not intrinsically motivating but range from behaviors learned through contingencies including external rewards of the environment to activities performed with a sense of volition because of their perceived value (Deci & Ryan, 2000). These motivation qualities vary intra-individually across different activities and contexts. The degree of internalization depends on the ability of an individual to cognitively and emotionally regulate their behavior. Therefore, young children might predominantly rely on more controlled qualities of regulation, whilst during development, reasons to engage in behavior may become increasingly internal (Deci & Ryan, 2000). Engaging in activities out of a sense of volition also depends on the context, specifically on the extent to which basic needs are supported (see Basic Needs Theory).

Many studies have examined the existence of the *internalization* *continuum* in a variety of contexts, including relationships, work, education, religion, prosocial behavior, parenting, psychotherapy, and health care (for an overview: Vansteenkiste et al., 2010). The manifestation of the covert growth tendency across this continuum is mostly researched by means of self-report questionnaires. This large number of studies shows that (i) the correlation patterns of the different motivation qualities reflect the proposed continuum with adjacent qualities being highly correlated, that is, extrinsic motivation correlates best with its neighbor quality of introjected motivation, and so on (Guttman, 1958; for an example: Ryan & Connell, 1989); and (ii) an individual’s dominant position on this continuum predicts various outcomes (Vansteenkiste et al., 2010). In general, higher motivation quality (more autonomous) can be linked with greater performance, persistence, social functioning, affect, and state of well-being over different contexts (Deci & Ryan, 2000, 2008). Therefore, increased motivation quality is a manifestation of the natural growth tendency (Figures 2 and 3).

### Basic Needs Theory

SDT argues that the human natural growth tendency—and with it the increase in motivation quality—depends on satisfaction of three *basic psychological needs*: Autonomy, Relatedness, and Competence (Deci & Ryan, 2008). *Autonomy* refers to being the perceived origin of one’s behavior (de Charms, 1968). A need for *Relatedness* refers to feeling connected with other individuals and the broader community (for review see: Baumeister & Leary, 1995; Ryan, 1995). *Competence* refers to feeling effective during interaction with the environment (Deci, 1975). Within SDT, these basic needs are viewed as universal and innate; they are not preferences learned through socialization (Vansteenkiste, Lens, Soenens, et al., 2006) (Figure 2). This implies that individuals benefit from basic need satisfaction, even when these needs are not valued consciously by themselves, their social context or culture, although cultural variation may exist in the intensity of these needs, and in the ways these needs are best satisfied (Deci & Ryan, 2002; Vansteenkiste et al., 2010). Moreover, satisfaction of basic needs supports the internal tendency toward growth (Figure 2).

A large number of studies has found that satisfaction of basic needs results in increased quality of motivation and well-being over different socio-economic groups, countries, and cultures (Deci & Ryan, 2008; Markland & Tobin, 2010; Vansteenkiste et al., 2010). A supportive, non-controlling interpersonal context in which individuals experience a full sense of choice and endorsement of their activity has been found to promote higher motivation quality in a wide variety of questionnaire-based and experimental studies (Deci et al., 1994; Vansteenkiste et al., 2010). For example, it has been shown that an autonomy-supportive versus controlling context enhances quality of motivation, and increases achievement and well-being as well (Vansteenkiste, Lens, & Deci, 2006). One practical example is described in the experiment of Murayama et al. (2015), where providing typically developing participants with a task-irrelevant choice (e.g., selection of task lay-out) resulted in increased performance as compared to a no-choice condition. This can be explained in terms of satisfaction of the basic need of autonomy, resulting in higher motivation quality. In contrast, controlling contexts have been associated with reduced learning, and maladjustment (Vansteenkiste, Lens, & Deci, 2006). Need-thwarting is associated with decreased motivation quality, which is accompanied with a decrease in positive feelings (interest and enjoyment) and a decrease in well-being. Prolonged need thwarthing may eventually lead to feelings of helplessness, rigid behavior patterns in search for (short-term) feelings of security, and externalizing problems (Ryan et al., 2016) (Figure 2).

### Goal Content Theory

*Pursuing goals* that provide experiences that satisfy basic psychological needs (autonomy, competence, relatedness) will facilitate the innate drive toward growth as compared to goals that thwart these needs (Kasser & Ryan, 1993). Such “intrinsic” goals refer to self-acceptance, affiliation, and community contribution (Deci & Ryan, 2000). These have intrinsic value because the focus is on developing inner potential. Achieving these intrinsic goals will support the basic needs, while with the pursuit of extrinsic goals, the attention is focused outside the person toward social comparison (Deci & Ryan, 2000). These are therefore not inherently satisfactory but depend on the reaction of others such as admiration of expensive property or acknowledgment of beauty. Moreover, pursuing intrinsic goals is another manifestation of the natural growth tendency (Figure 2).

This theory has been confirmed over different cultures (for a review: Vansteenkiste et al., 2010). Intriguingly, extrinsic goals have been shown to lead to lowered well-being even when the individual, and his/her social context, seemed to endorse and prefer extrinsic goals attainment (Vansteenkiste et al., 2008). Pursuit and/or attainment of intrinsic, compared to extrinsic, goals has been positively related to personal well-being, and to health-related, interpersonal and societal outcomes (Vansteenkiste et al., 2010) including less substance use, less romantic relationship conflict, and smaller ecological footprint (for a review: Vansteenkiste et al., 2008). Indeed, framing everyday activities such as sorting out garbage or doing sports in terms of intrinsic versus extrinsic goals is linked to increased performance and well-being (Vansteenkiste et al., 2004). In general, valuing, pursuing, or fulfilling a personal goal is associated with positive feelings. However, the SDT specifically posits that this is fully dependent on goal content (Kasser & Ryan, 1996). The SDT assumes an interaction between the environment and the innate growth process (Vansteenkiste & Sheldon, 2006). Hereby it categorizes environmental influences as positive or negative (in accordance or not with this growth tendency) (Vansteenkiste & Soenens, 2009).

### Causality Orientations Theory

Motivation quality depends on specific life contexts or situations. For example, a student may study mainly to attain his/her parents’ approval, except for history, as the enthusiastic stories of the teacher fuel the student’s interest and imagination (Vallerand, 1997). In addition to such within-subject context effects, inter-individual differences in orientation toward a behavior regulation strategy exist (Deci & Ryan, 1985b). Some individuals regulate their behavior based on personal value and interest (autonomous orientation), others more as a function of external pressure (controlled orientation), and some do not perceive events as in their control (impersonal orientation) (Deci & Ryan, 1985b). Thus, each of these orientations exist intra-individually, and due to experiences one of them can be more dominant in an individual, influencing one’s perception and interpretation of the environment (Vansteenkiste et al., 2010). Therefore, we can assume that (groups of) individuals have a specific motivational profile incorporating motivational orientation over different situations and tasks (Vansteenkiste & Mouratidis, 2016). Moreover, a tendency toward autonomous orientation is an additional manifestation of the natural growth tendency (Figure 2).

Questionnaire-based research showed that an individual’s orientation toward more autonomous versus controlled processing is associated with a more open, sincere, and accurate attitude during social interaction (Hodgins & Knee, 2002). For example, it was found that autonomy-orientation was associated with an information-oriented style in the process of identity exploration as opposed to a normative or diffuse-avoidant style (Soenens et al., 2005). In addition, experimental research showed that priming participants with autonomy- versus control-related words during a task resulted in increased enjoyment, increased performance, and elevated self-worth (Hodgins et al., 2006, 2007; Levesque & Pelletier, 2003). A continuous transactional process can be assumed, where an autonomous orientation might provide more opportunities for potential basic need satisfaction resulting in increased enjoyment or performance during tasks or activities. This experience might in turn strengthen autonomous orientation.

### Cognitive Evaluation Theory

While sometimes external contingency can increase performance, there is substantial evidence that addition of an external incentive such as monetary rewards can negatively influence intrinsic motivation, performance, affect, frustration, and well-being for relatively fun or interesting tasks (for reviews: Cerasoli et al., 2014; Deci et al., 1999a). These phenomena are sometimes referred to as “the hidden costs of rewards.” This *undermining effect* of an external incentive on intrinsic motivation presents itself as a decline in behavior persistence lower than baseline level, when an external incentive is added and subsequently withdrawn (Deci et al., 1999a; Murayama et al., 2010). The undermining effect is more likely to occur when; (i) the task is experienced as relatively fun or interesting, (ii) performance quantity rather than quality is emphasized, (iii) the incentive is contingent on performance and is experienced as controlling rather than informative (for a review: Cameron & Pierce, 1994; Cerasoli et al., 2014; Deci et al., 1999a). Here, the SDT provides the following explanation: that the addition of an external incentive shifts the perceived locus of control from voluntary internal reasons to external control (de Charms, 1968) resulting in a reduction in motivational quality (Deci, 1976). This explanation relates to the overjustification hypothesis (Lepper, 1981). This hypothesis states that when a behavior draws on both intrinsic (e.g., fun activity) and extrinsic motivation (e.g., financial reward), the individual will attribute the behavior only to the more salient external contingency. This is because this extrinsic reason alone is already sufficient to justify the execution of the behavior (Lepper et al., 1973). There has been debate about the undermining effect of external incentives on intrinsic motivation (e.g., Deci et al., 1999b; Lepper et al., 1996) and meta-analyses have documented the effects well. The undermining effect presents under specific conditions, such as when the task is experienced as intrinsically motivated (else there is no intrinsic motivation to be undermined), when the reward is not unexpected, and when it is tangible: Deci et al. (1999a) reviewed 128 studies that had examined the undermining effect of external rewards on intrinsic motivation and found that engagement-contingent, completion-contingent, and performance-contingent rewards all undermined free-choice intrinsic motivation, and that verbal positive feedback *enhanced* intrinsic motivation. The perspective of the frustration theory may also explain this undermining effect. It can be argued that the above-referenced decline in behavior persistence lower than baseline may reflect a frustrated reaction based on loss of an anticipated reward (Amsel, 1958; Douglas & Parry, 1994). In other words, after first having engaged in a task with reinforcers, and after that being given the opportunity to engage in the task out of free will but without reinforcers, the frustration about the lack of reinforcement after having been reinforced may be a reason to not want to engage in the non-reinforced task. Interestingly, participants with ADHD seem to become more frustrated than typically developing controls when anticipated rewards fail to appear (Douglas, 1985).

Lepper et al. (1973) showed that children who initially took pleasure in drawing were less motivated to re-engage in drawing during a post-test free-choice period if they had previously attained a material reward for doing so, as compared to children who had not received a reward. In another example, Warneken and Tomasello (2013) demonstrated that previously presented altruistic tendencies decrease in young children when coupled with an external material and verbal reward, compared to a non-rewarded group.

In conclusion, SDT offers a broad perspective on motivation where individuals have a natural tendency toward growth and development. This internal propensity is manifest, and thus measurable, in four ways: (i) intrinsic motivation (e.g., experiencing activity as fun and interesting), (ii) internalization of drives (degree of autonomous vs. controlled motivation quality the individual exhibits), (iii) experienced basic need satisfaction/frustration, and (iv) pursuit of intrinsic goals. These internal motives mediate the relationship between the context on the one hand, and motivated behavior and a variety of life outcomes on the other hand (Figure 1). Extensive SDT-based research has used a variety of measures such as behavioral *outcomes, engagement*, *self-report*, and more recently also *neuro-imaging* (e.g., Murayama et al., 2010, 2015). Although the SDT allows for formulation of specific hypotheses concerning motivation in individuals with ADHD, current ADHD research has not, or only very limitedly, adopted the theory. Additionally, in the next paragraphs we will demonstrate that current ADHD theories and research generally bypass internal motives and generally only study the effect of external triggers on motivated action including *preferences* and *performance* using behavioral, physiological, and neural measures.

## Theories and Research of Motivation in ADHD

ADHD is a pervasive and persistent developmental disorder characterized by age-inappropriate levels of inattention, hyperactivity, and impulsivity (American Psychiatric Association, 2013). Aside from cognitive impairments, motivational alterations are described as underlying deficits (e.g., Luman et al., 2010; Plamondon & Martinussen, 2019). Theoretical models of ADHD that include motivation, vary in their acknowledgment of internal motives. Some seem to focus on the effect of external triggers on motivated action, without (explicitly) formulating hypotheses about the mediating role of internal motives, while others have incorporated internal motives. However, whether explicitly described or not, all models do *implicitly* include internal motives. Below, the most prominent ADHD theories that touch on motivation will be briefly explained, and situated on the different levels of Reeve’s motivation model (Figure 1).

Generally, theoretical models explain motivational alterations in ADHD in terms of a dysregulated dopamine system which results in (i) an altered sensitivity to reward and/or punishment (Barkley, 1997; Sagvolden, 2000; Sergeant, 2000; Sonuga-Barke, 2002; Tripp & Wickens, 2009) and (ii) difficulty regulating arousal state (Sergeant, 2000, 2005). Related studies mostly utilize choice/preference, task performance, or altered physiological/brain function as outcomes. In Reeve’s (2012) model these outcomes are situated in the link between *social context/external triggers* and *motivated action* (Figure 1) (see for review Luman et al., 2010). In what follows we will discuss ADHD theories that are to some extent explicit in their description of what in SDT terms would be called “internal motives” that are at play in ADHD: (1) the Dual Pathway Model (Sonuga-Barke, 2002), (2) the Unifying Theory (Barkley, 1997), and (3) the Cognitive-Energetic Model (Sergeant, 2000, 2005).

### Theories

#### The dual pathway model

**Figure 4.**
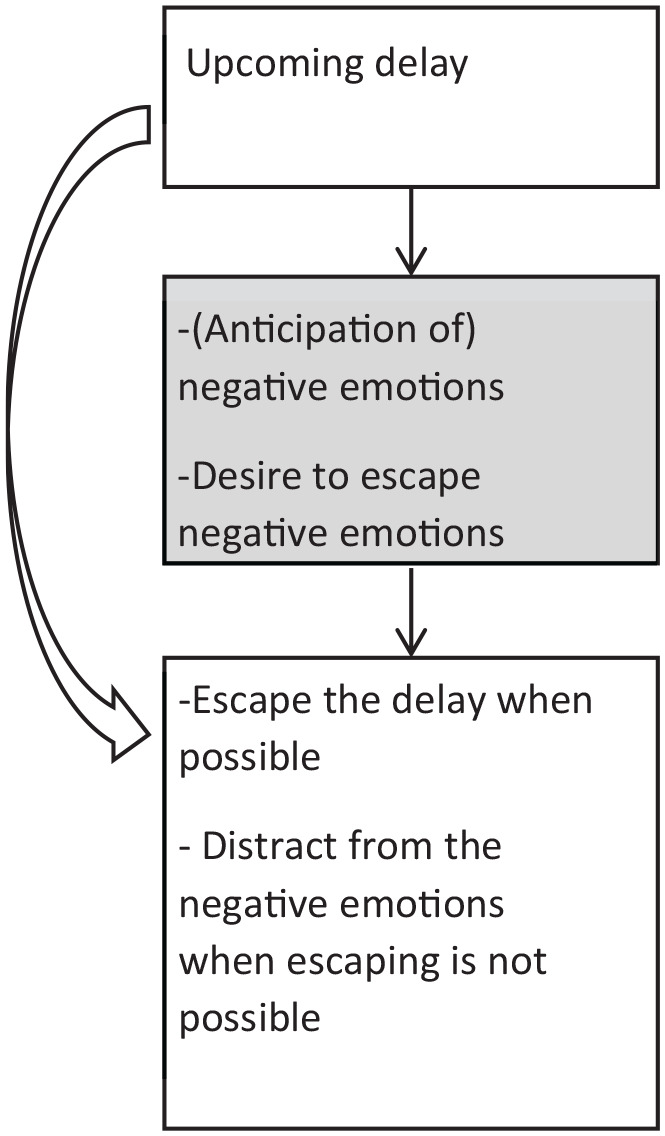
Visualization of the Delay Aversion hypothesis (Sonuga-Barke, 2002). The internal motives (grey box) are hypothesized to mediate the relation between environmental factors (upcoming delay) and outcomes (behavior). Note that empirical research so far is largely limited to (arrow) studying the link between the external factor (presenting choices between smaller sooner and larger delayed rewards) and the behavioral outcome (choosing to escape the delay). The role of internal motives deserves more attention.

The Dual Pathway Model (Sonuga-Barke, 2002) is inspired by Sagvolden’s dopamine dysfunction related Dynamic Developmental Theory (DDT) of ADHD (Sagvolden, 2000; Sagvolden et al., 2005). This DDT is rooted in an animal model of ADHD and hypothesizes that altered dopamine function, in interaction with environmental factors, results in altered reinforcement of behavior, which in turn leads to delay aversion and ADHD symptoms. The Dual Pathway Model (Sonuga-Barke, 2002) hypothesizes that there are two pathways leading to ADHD symptoms: (1) an executive function pathway, in which brain-related processes that are relevant for executive functions contribute to relatively weak executive functions such as inhibitory control, which form the basis for the ADHD symptoms as they are observed in daily life; and (2) a delay aversion pathway, in which brain-related processes that are relevant to motivational functioning contribute to delay aversion, which forms the basis for behaviors that are motivated by the desire to escape delays, and express as the ADHD symptoms as we observe them in daily life. The delay aversion pathway can be viewed as a motivational pathway, in that delay aversion is a motivational style, and has to do with preferences (for smaller immediate rewards), rather than with (dis)abilities. In Reeve’s terms, the dual pathway model, and in particular the motivational delay aversion pathway, provides specific predictions about *Internal motives* that mediate the relation between *External triggers* and *Motivated action* (Sonuga-Barke, 2002). Sonuga-Barke (2002) hypothesized that individuals with ADHD develop an aversion to delay, that is waiting, over time. The key notion is that negative emotions experienced during waiting (Reeve’s “*Internal motives*”) result in attempts to escape such delays when possible (*Motivated action*), and in strategies to distract from these negative emotions when escaping is not possible (*Motivated action*) (see Figure 4).

Empirical support for this theory is growing but note that most of the evidence is limited to data at the level of the outcome (*Motivated action*) and not the negative emotions experienced during waiting (internal motives). One strand of studies supporting this model focuses on the increased preference/choice for small immediate rewards over larger delayed rewards in individuals with ADHD compared to their typically developing peers. More specifically, these studies show consistently and strongly (with a medium effect size) that individuals with ADHD, when presented with choices between smaller immediate and larger delayed rewards, choose the smaller immediate rewards more often than individuals without ADHD (Jackson & MacKillop, 2016; Patros et al., 2016). Moreover, Scheres et al. (2013) found that this increased preference for small immediate rewards was more strongly linked with self-reported difficulty for waiting in individuals with ADHD compared to typically developing peers. The dependent variable in these empirical studies (preference/choice) can be considered an outcome variable at the behavioral level, but it does not inform us on how the internal motives (negative emotions) have led to this preference (Figures 1 and 4), with the exception of self-reported difficulty during waiting in the Scheres study (Scheres et al., 2013).

Furthermore, a small number of studies provided evidence at the brain level for this model. First, Lemiere et al. (2012) showed that adolescents with ADHD had exaggerated brain responses in the amygdala, ventral striatum, and orbito-frontal cortex (areas related to emotional processing) to cues signaling unescapable delays compared to cues signaling escapable delays. Second, in a study of Wilbertz et al. (2013) adults with ADHD had different amygdala responses to increasing delays, and increased skin conductance compared to controls, which can be interpreted as a stronger emotional response. Third, Van Dessel et al. (2017) found that individuals with ADHD showed amygdala and prefrontal cortex hyperactivation for cues signaling certain delay as opposed to no delay. Additionally, it was shown that this hyperactivation mediated the relationship between ADHD group membership and delay aversion in daily life. Fourth, Mies et al. (2018) demonstrated that adolescents with ADHD had stronger preferences for small immediate rewards over larger delayed ones than controls, and that those individuals with ADHD who were delay averse in daily life, showed a stronger association between increasing delay durations and increasing amygdala activation. These studies are a start in examining the internal motives such as negative emotions that mediate the link between ADHD symptoms and motivated action such as preferred choice for small immediate rewards (see Hsu et al., 2015; Lemiere et al., 2012; Scheres et al., 2013; Wilbertz et al., 2013 for some initial work on this).

#### The unifying theory

Although the core of Barkley’s (1997) Unifying Theory is about executive dysfunction, Barkley generated several hypotheses that are relevant in the context of motivation. Specifically, Barkley proposes that individuals with ADHD are delayed in executive function development and may remain impaired in the internalization of self-directed speech. More specifically, Barkley suggests that the relative lack of self-directed speech in individuals with ADHD makes it hard to maintain goal-directed behavior in the context of tedious tasks. Therefore, Barkley hypothesizes that individuals with ADHD may remain more dependent on external reinforcers to maintain goal-directed behaviors for tasks that are not intrinsically motivating compared to their peers. This self-directed speech could be situated at the level of *Engagement* within *Motivated action* (Figure 1).

#### The cognitive energetic model

A third important and relevant theoretical model of ADHD that is relevant in the context of motivation is the *Cognitive Energetic Model* (Sergeant, 2000, 2005). In this model, Sergeant proposed, among other notions, that individuals with ADHD have difficulties in recruiting or allocating effort. Within this framework, effort is described as a management system that regulates arousal/activation levels needed to meet task demands, which in turn influences information processing. According to the model’s predictions in terms of individuals with ADHD, weak effort allocation leads to poor regulation of arousal/activation levels which in turn results in poor information processing. This results in ADHD-related inattentive, impulsive, and overactive behavior. In Reeve’s (2014) framework, effort is viewed as one behavioral expression of motivation, and therefore, lower applied effort could be a reflection of lower motivation in individuals with ADHD. However, within the Cognitive Energetic Model, effort is not so much described as a manifestation of motivation, but rather as a cognitive control/executive function. More recently, effort has been operationalized as a subjective experience, in which cognitive control and motivation both form important elements (e.g., Westbrook & Braver, 2015). The Cognitive Energetic Model can be placed at the level of *Behavioral* *expression* of *Motivated action* (Figure 1).

### Relevant Research of Motivation in ADHD

#### Early work

First, a body of early empirical work by Douglas on motivational factors in understanding ADHD has been very influential. Her studies highlighted the importance of sustaining effort and motivation in individuals with ADHD, especially during long tasks and with minimal reinforcement (Douglas, 1972; Douglas & Parry, 1983, 1994). Additionally, she has suggested that individuals with ADHD demonstrate relatively quick habituation to effects of reward (Douglas, 1999). Interestingly, Douglas’ work has also shown that behavior/performance of individuals with ADHD significantly improves when tasks are made to be more salient, novel, interesting, or stimulating (Douglas, 1985; see also Zentall, 1986; Zentall & Shaw, 1980). This early work by Douglas mostly includes environmental effects on behavior and performance only (*Motivated action* and *Life outcomes*; Figure 1), although Douglas (1985) does explicitly state that children with ADHD experience a lack in intrinsic motivation.

#### Effects of reinforcers

Secondly, Luman et al. (2005) published a review paper on the effects of reinforcement contingencies on ADHD. This review demonstrated that reinforcers (both reward and response cost) improved performance, in children with ADHD and in controls, although the improvement was more prominent in those with ADHD (outcomes can be situated at level of *Motivated action*). Similarly, but more specifically focused on the executive function Inhibitory Control, Ma et al. (2016) demonstrated in a meta-analysis that reinforcement can normalize inhibitory control in children and adolescents with ADHD to the baseline level of controls. Furthermore, the data suggests that inhibitory control may improve to a larger extent in youth with ADHD compared with controls, as a function of reinforcement. Within these reviews/meta-analyses, only a minority of studies included data on self-reported and observed motivation aside from the effect of reinforcement on task performance. In these few studies (Carlson et al., 2002; McInerney & Kerns, 2003), motivation as it was observed by the experimenters improved as a function of reinforcement, while self-reported motivation (level of enjoyment) did not.

#### Brain studies

On the brain level, a growing body of functional Magnetic Resonance Imaging (fMRI) research has shown that compared to typically developing peers, individuals with ADHD demonstrate a medium-sized reduction in striatum activation when anticipating money that is offered contingent on performance (see for a meta-analysis Plichta & Scheres, 2014). This finding refers to brain activity as one expression of *Motivated action* (Figure 1).

#### Motivation beyond the effects of reinforcers

While still limited, there is some research on internal drive or intrinsic motivation in the context of ADHD, playing at the levels of internal processes/motives (Figure 1). First, some research tried to explain the link between ADHD and decreased academic achievement, in terms of a different pattern of achievement motivation (Achievement Goal Theory; Barron et al., 2006). Second, while aiming to better understand the relatively poor academic performance of individuals with ADHD, Carlson et al. (2002) found that children with ADHD and their teachers reported ADHD-specific motivational alterations such as decreased academic motivation (although this result was specific for the inattentive type of ADHD), more reliance on external feedback than internal standards to evaluate performance and dependence on teacher involvement to complete academic work. An influential review of Smith and Langberg (2018) finds strong evidence for lower academic-related motivation associated with ADHD in youth, and motivation played a role in academic outcomes, especially in reading. Note, however, that a study in college students (Reaser et al., 2007) found that, while students with ADHD had lower scores on the LASSI subscale Motivation, their scores on the subscale Attitude did not differ from the reference group. The authors suggested that while students with ADHD’s motivation to attend to specific tasks may be low, they did not demonstrate a particular difficulty with being interested in school. We suggest here that findings may vary depending on which instrument is used/which vary aspect of academic motivation is being measured, and it is also possible that college students with ADHD represent a subgroup of individuals with ADHD who are relatively high in motivation/interest for school in general. Third, in a qualitative study (interviews), children and adolescents with ADHD reported being motivated by similar tasks and activities as peers. Worth mentioning is that in this study, children and adolescents with ADHD spontaneously referred to the three basic SDT needs of Autonomy, Relatedness, and Competence when prompted to report what motivated them in everyday life (Morsink et al., 2017). Finally, Morsink et al. (2019) did a quantitative study in which children with ADHD and peers completed questionnaires about what kind of tasks and activities they typically look forward to, and which tasks motivate them. This study showed that children and adolescents with ADHD reported being equally motivated as typically developing peers to engage in a task when the following task characteristics were present: rewarded, competitive, socially evaluated, and containing predictable elements, although they mentioned to be less motivated than typically developing children and adolescents to engage in tasks with these characteristics; cognitively challenging, requiring focus, collaborative, marked/graded (Morsink et al., 2019).

#### Concluding thoughts

**Figure 5.**
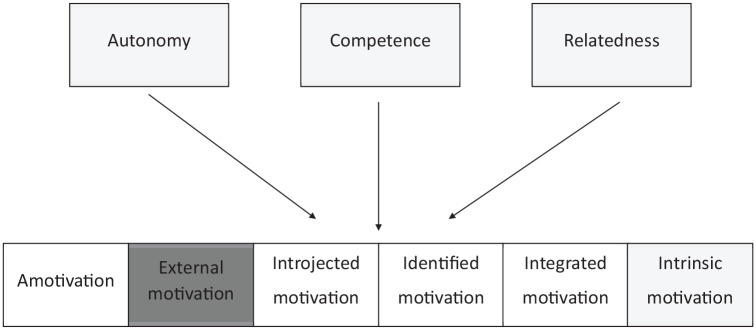
Simplified overview of which parts of SDT have been studied in ADHD research. The darker the color, the more it has been studied in ADHD research.

In sum, theories and work on the role that motivation plays in individuals with ADHD have resulted in a significant body of literature giving insight into the effects of reinforcers on performance, behavior, and the brain. This important body of literature can further benefit from a broader theoretical framework of motivation such as SDT, appreciating the role that internal motives play, focusing on the full spectrum of motivation qualities ranging from intrinsic to extrinsic, and using a broader set of measures that can give a fuller picture of motivation (see Figure 5). Thus, while effects of, especially monetary, reinforcers on task performance are well studied in individuals with ADHD, there is a relative lack of research including measures of internal motives, which according to the SDT form the basis of directed behavior (Deci & Ryan, 2000; Reeve, 2014).

## How Can SDT Inform Further Research on Motivation in ADHD?

Based on the above, we suggest that SDT can provide a useful framework within which our understanding of the role which motivation potentially plays in the context of ADHD can be broadened and enriched. Each of the SDT’s mini theories can provide useful ways to inform future ADHD research.

### Organismic Integration Theory

Relating to motivation qualities, what is currently proposed in ADHD theories is that individuals with ADHD have an innate or learned altered reaction toward external rewards (Sagvolden et al., 2005; Sonuga-Barke, 2002; Tripp & Wickens, 2009), where they seem to need rewards in order to reach a comparable performance level as their peers, and where they have a stronger preference for immediate rewards than their peers (Jackson & MacKillop, 2016; Luman et al., 2005, 2010; Ma et al., 2016; Patros et al., 2016). As the SDT illustrates a continuum of *motivation qualities* ranging from a-motivation to intrinsic motivation, we suggest that it is important and relevant to broaden our focus to the full continuum of motivation qualities in the study of ADHD, as our knowledge about the internalization process of motivation in individuals with ADHD is limited. Therefore, rather than studying controlled motivation, also autonomous/voluntary motivation (see Figures 2 and 3) is relevant to be studied in individuals with ADHD. For example, while individuals with ADHD are suggested to be “less motivated” (e.g., Barkley, 1997; Volkow et al., 2011), especially for cognitively challenging (Morsink et al., 2017) or academic tasks (Langberg et al., 2013), research addressing this hypothesis regarding more autonomous forms of motivation is relatively limited. While Carlson et al. (2000, 2002) made an early start with this by studying intrinsic motivation for academic tasks (Carlson & Tamm, 2000; see above), more work is needed. In order to learn more about motivation qualities in individuals with ADHD, study outcomes have to go beyond performance and behavior on academic (and often not-liked tasks) but may also include more direct measures of (self-reported) motivation, engagement during free choice paradigms or task experience, or well-being during questionnaire-based research, and research that includes intrinsically motivating tasks.

**Figure 6.**
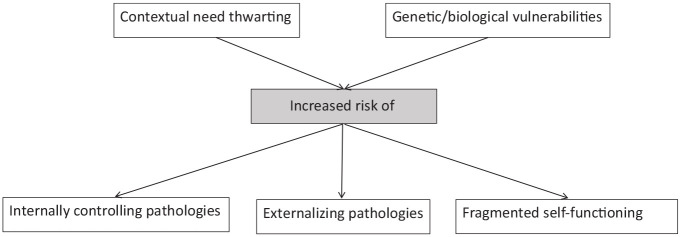
Link between need thwarting (of Autonomy, Competence and Relatedness) and externalizing pathologies according to SDT. Adapted from Ryan et al. (2016). Copyright 2015 John Wiley & Sons, Inc.

A relevant question arising from organismic integration theory is whether the internalization/socialization of values, as part of the continuum in the SDT model, is related to ADHD symptoms. Vansteenkiste and Ryan (2013) describe various cross-sectional and longitudinal studies that illustrate how thwarting of the basic need autonomy can play a role in psychopathology, through hampering in internalization of values and socializations, characterized by difficulties in self-regulation of behaviors (Figure 6). In other words, when autonomy is structurally thwarted, this may prevent individuals from developing a sense of volition (this sense of volition characterizes autonomous qualities of motivation) to engage in certain behaviors or tasks. In the case of ADHD, we could ask the following question: Do individuals with ADHD develop more slowly in terms of internalization/socialization of values (this may be predicted based on Furukawa et al., 2019), which in turn may contribute to the environment relying on external reinforcers to control behavior, situating individuals with ADHD more toward the controlled end of the motivation continuum as compared to their typically developing peers? It may be hypothesized that individuals with ADHD experience less voluntary and more controlled qualities of motivation, potentially as a result of their increased reliance on external guidance. Whether individuals with ADHD might experience this external guidance as pressure or in line with their own beliefs is an empirical question. Longitudinal research will be an important design to apply to questions such as these.

### Basic Needs Theory

Given that SDT describes motivation as consisting of a dimension of motivation qualities fueled by basic needs of Autonomy, Competence, and Relatedness, it may be worth investigating to what extent Autonomy, Competence, and Relatedness are supported versus thwarted in individuals with ADHD by their environment and what effect this has on the development of their ADHD and associated symptoms longitudinally. Vice versa, it may be worth investigating which effects ADHD symptoms have on the extent of basic need support offered by parents/teachers. Based on the findings that need-thwarting is related to a range of psychopathological behaviors including externalizing behavioral problems (Ryan et al., 2015), and based on studies that show that caregivers use more controlling styles in relation to children with ADHD (Reeve, 2009), one may hypothesize that children with ADHD experience relatively high levels of need thwarting and/or relatively low levels of need support. Along the same lines, it may be suggested that when these individuals’ basic needs are supported, their externalizing problems might decrease.

Specifically, it would be interesting to follow-up on Rogers and Tannock’s (2018) research on the relationship between ADHD symptoms and decreased *basic need satisfaction* and increased *basic need frustration* in everyday life. In order to better understand the nature of this association, a study with a longitudinal design and measures of context of the child (i.e., need thwarting/supporting of the teacher) could be a first step. A first hypothesis might be that ADHD symptoms and related difficulties such as EF dysfunctions and externalizing problems might *elicit* a more controlling style from the teacher (Reeve, 2014), whilst children and adolescents with ADHD may benefit from a basic need supporting context in a similar way as their typically developing peers (Grolnick, 2002; Grusec & Goodnow, 1994; Morsink et al., in preparation ). This may lead to a mismatch between the children’s needs and the teacher style, and may possibly exacerbate the symptoms in the child, which in turn may lead to a further increase in controlling teacher style. If indeed such a dynamic interaction between teacher style and child symptoms would be demonstrated, this has implications for interventions. A second hypothesis might be that teachers (and parents) may provide opportunities for basic need satisfaction in the same way for children and adolescents with ADHD as their peers, but that this is not *experienced* in the same way by individuals with ADHD as it is by controls. After all, in the study by Rogers and Tannock, basic need satisfaction was measured by asking children how they perceived the need satisfaction by their teacher. For example, due to a basic learning deficit/attention problems they might not pick up these supporting signals from their environment similarly as TD children (see De Meyer et al., 2019), which may lead to children with ADHD not feeling supported in their basic needs despite their teacher’s/parents’ efforts.

A third hypothesis might be that children and youngsters with ADHD might put more strain on their caregivers because of their more challenging behavior. This may interfere with the caregiver’s basic need satisfaction, which may hamper their ability to implement autonomy-supportive strategies to the child (de Haan et al., 2013), which in turn may lead to an exacerbation of ADHD symptoms. A study by Tannock and colleagues (Rogers et al., 2009) suggests that such processes may indeed be at play, as fathers of children with ADHD “used academic pressure in response to their child’s ADHD behaviors, thus perpetuating a cycle of family stress and negative interactions” (p. 179).

A fourth hypothesis may be that providing support of the basic needs to children with ADHD may have a positive effect on their ADHD symptoms and related functioning such as academic achievement. Relevant to this is a study by Hamre and Pianta (2005) who showed in a prospective study that children who were identified as at-risk children (in terms of behavior, attention, social, and academic) at a young age and were then later placed in a classroom with a teacher who provided emotional support, including autonomy support as reflected by being tuned into the child’s needs, moods, interests, and capabilities had higher achievement scores and less conflict with teacher than at-risk children who were then placed in less supportive classrooms. A recent longitudinal study provided support for the notion that parental autonomy support predicts later child behavior, but this was independent of ADHD symptoms: children, including those with ADHD, who received more parental autonomy support at the age of four to five were more likely to show a reduction in problem behaviors, such as bullying, over the next 4 years, than children who received less autonomy support at that early age (Rajendran et al., 2016). Further longitudinal research in ADHD is needed to follow up on this, and to determine whether basic need support provided by parents and/or teachers may alleviate ADHD symptoms and improve their (academic) functioning and wellbeing.

In sum, there is some initial evidence of a relation between basic psychological needs frustration and symptoms of ADHD (and related outcomes such as academic achievement). What the causal direction of this relation is, will need to be the focus of future research. Likely, there is a bidirectional relation that dynamically develops over time. Moreover, if ADHD is indeed associated with decreased basic need satisfaction, ways in which the basic needs of individuals with ADHD can be effectively supported by their parents and teachers should be assessed as well, and this may have implications for interventions (see below).

### Goal Content Theory

It is an empirical question whether individuals with ADHD prefer different goals than individuals without ADHD. May extrinsic goals—such as wealth and fame—be preferred over intrinsic ones—such as self-acceptance, affiliation, and community contribution—to a larger degree in those with ADHD as compared to typically developing individuals? And would (the pursuit of) the experience of self-acceptance, affiliation, and community contribution be associated with decreased externalizing behavior in individuals with ADHD?

Of particular interest may be to study motivation of individuals with ADHD not only in the academic domain, but across life domains. Motivation in the academic domain has been studied extensively. An influential review of Smith and Langberg (2018) finds strong evidence for lower academic-related motivation associated with ADHD, and points out that these results are scarcely evaluated in the light of broader theoretical and empirical motivation literature, underlining the need for a comprehensive theoretical framework on motivation that can be used as a context within which relevant research questions for individuals with ADHD can be formulated, and empirical findings interpreted. Extending the study of motivation beyond the academic domain, we may want to focus on relevant domains such as health care (Ng et al., 2012), professional (Rigby & Ryan, 2018), spare time (Standage & Ryan, 2012) contexts, while using SDT-related questionnaires. The specific themes mentioned in qualitative research by Morsink et al. (2017) that were reported by youngsters with ADHD could serve as a useful starting point for follow-up quantitative research.

We believe it is unlikely that individuals with ADHD would have low intrinsic motivation in general, across life domains and contexts. It seems more probable that individuals with ADHD, like individuals without ADHD, will experience high intrinsic motivation for certain activities and low intrinsic motivation for others. For example, Sibley and Yeguez (2018) showed in a qualitative study that individuals with ADHD experienced intrinsic motivation during high interest subject matter and vocational activities as well as in environments in which high levels of autonomy were experienced, and that engaged the individual’s strengths. Such in-depth (qualitative) investigations (Morsink et al., 2017; Sibley & Yeguez, 2018) which have been able to identify tasks and activities that are experienced as intrinsically motivating by individuals with ADHD could inform and be followed by quantitative research that could collect data resulting in a “motivation quality profile.” Moreover, to follow-up on research of Morsink et al. (in preparation ) it would be interesting to examine for which task types/elements individuals with ADHD have decreased motivation, that is those that include delay (Sonuga-Barke, 2002), that lack external rewards (Luman et al., 2005), that require adjustments in the arousal system (Sagvolden, 2000), or do not result in basic need satisfaction (Deci & Ryan, 2000). Related to this suggestion, we believe it would be interesting to study goal-directed behavior in relation to specific task characteristics that range in interest and goal content. We expect that Barkley’s hypothesis that ADHD is related to a lower internal drive to maintain goal-directed behavior may be confirmed for uninteresting and boring tasks but not for tasks that are experienced as intrinsically motivating. This is based on research showing that individuals with ADHD experience low motivation for academic tasks (Smith & Langberg, 2018), and on research showing that youngsters with ADHD show greater motivation for, and benefit more from working memory training when it is presented as a fun game instead of in its regular training format (Prins et al., 2011). Future research can address this by comparing tasks that are intrinsically motivating for individuals with ADHD with tasks that are not intrinsically motivating and test whether individuals with ADHD, more than controls, depend on external reinforcers.

Moreover, longitudinal studies can elucidate if children and adolescents with ADHD experience a developmental delay in goal directed behaviors. Finally, the association between motivation and goal-directed behavior may also be reversed, with goal-directed behavior potentially having an influence on motivation. For example, Sibley and Yeguez (2018) showed that individuals with ADHD identified, among others, goal formulation as a strategy to enhance motivation. The (bidirectional) relation between goal-directed behavior and motivation needs to be studied in those with ADHD in longitudinal research.

### Causality Orientations Theory

As some groups of individuals possess an orientation toward more autonomous or controlled regulation strategies (Deci & Ryan, 1985b), it might be relevant to assess whether individuals with ADHD exhibit a specific motivational orientation style either globally or in relation to specific situations. Furthermore, would this style be characterized by controlled orientation, that is more under influence of external as opposed to internal drives? If individuals have a decreased self-directed speech, they might on average regulate their behavior predominantly based on controlled rather than autonomous strategies. Alternatively, ADHD symptoms lead these individuals to be more dependent on parental control which may hamper the child’s opportunities to further internalize their motivation over time. Also, it will be interesting to study which role parenting style and academic context (controlling vs. autonomy-supportive) may play herein. It will be challenging to determine causality between such factors (see above). Longitudinal research designs will help address such questions.

### Cognitive Evaluation Theory

The focus on external reinforcement to promote desired behaviors might come with potential costs to performance quality, motivation quality, affect, and well-being. The undermining effect of extrinsic reinforcers on intrinsic motivation has been demonstrated in typically developing individuals particularly under certain circumstances such as when the reinforcer is experienced as controlling rather than as informative, and when the motivation quality for that task or activity is reasonably high to begin with (Cerasoli et al., 2014). However, this has scarcely been studied in individuals with ADHD and the limited studies failed to identify an undermining effect in individuals with ADHD and their typically developing peers (Carlson & Tamm, 2000; Carlson et al., 2000). These authors suggested that the conditions in which the undermining effect might occur need to be elucidated further with additional measurements of motivation (performance, self-reported motivation, behavioral engagement). Also they suggest that alternative tasks are needed in order to firmly establish whether or not external reinforcers may undermine intrinsic motivation in individuals with ADHD differentially as compared to controls. The current research on motivation in ADHD shows increased performance during reinforcement (such as reviews of Luman et al., 2005; Ma et al., 2016; see below).

However, the studies included in these reviews consistently rely on typical cognitive laboratory tasks such as arithmetic tasks, continuous performance tests, paired associate memory tasks, repetitive motor tasks, executive functions tasks (Luman et al., 2005; Ma et al., 2016; Smith & Langberg, 2018), and use performance as the only or main dependent variable, which is not an optimal measure for intrinsic motivation. These tasks can be described as repetitive, lengthy, difficult, and boring, all factors that are suggested to minimize intrinsic motivation especially for individuals with ADHD (Morsink et al., 2017). Also, these are tasks that we expect individuals with ADHD to perform poorly on. Importantly, SDT theorists argue that for the undermining effect to occur, the task or activity needs to be perceived as relatively interesting or useful to begin with, otherwise there is not much intrinsic motivation to be undermined (Murayama et al., 2010). Douglas (1985) already showed the importance of introducing relatively interesting tasks to investigate ADHD-related motivational alterations alongside research using typical executive function tasks. For example, Prins et al. (2011) demonstrated that children with ADHD chose to spend more time training their executive functions in game-versions versus non-game versions of such tasks. Thus, we will need to select tasks that are to some extent interesting, meaningful, or enjoyable to the participants. Only then can we fully appreciate whether or not there are hidden costs of rewards in individuals with ADHD. We must keep in mind that the undermining effect might present itself differently in individuals with ADHD compared to controls: Maybe for individuals with ADHD offering performance-contingent rewards helps them to keep long-term goals more online, and aids in directing and maintaining goal-directed behaviors.

Additionally, SDT-focused experiments, as opposed to ADHD studies, typically take into account a range of task-related outcomes besides post-task behavioral persistence, such as performance quality (i.e., deep learning), level of intrinsic motivation, affect, and state well-being during and after the task. All of these seem to decrease when extrinsic incentives are included in the research design (Cerasoli et al., 2014). Therefore, it will be interesting and relevant to include similar outcome variables in ADHD research as well. While in some occasions, reinforcers may positively influence the targeted behavior or performance, other outcome measures such as intrinsic motivation, affect, and wellbeing may suffer in individuals with ADHD, and potentially to a larger extent than in individuals without ADHD. In sum, we suggest that we further study these potential costs of using external reinforcers by including other relevant outcomes measures will be included in addition to intrinsic motivation, such as wellbeing and self-esteem, and by doing so, we can start to have a fuller understanding of both the positive and negative effects that reinforcers may exert in individuals with ADHD.

### SDT as Mediational Model

The SDT theory can be simplified in a mediational model where *Internal motives*, that is motivation quality and underlying basic need satisfaction, mediate the effect of *External triggers* such as parental support on *Motivated action* and *Life outcomes* (Figure 1). Frielink et al. (2018) have tested this mediational model in a group of adults with intellectual impairment, and found that autonomous motivation and need satisfaction statistically mediated the association between autonomy support and well-being. The authors concluded that “the self-determination theory provides insights relevant for improving support for people with intellectual disability.” In similar ways, this model may suggest specific ways in which the environment can aid individuals with ADHD to increase their motivation, performance, and well-being.

### Supplementing Current ADHD Research With Measures of Internal Motives

Finally, we think that current research on motivation in individuals with ADHD can greatly benefit from the inclusion of measures relating to internal motives. For example, research testing the delay aversion hypothesis (dual pathway model) makes use of choice paradigms in which participants choose between smaller immediate rewards and larger delayed ones. Adding measures of individuals’ experiences during waiting such as skin conductance, heart rate, pupil dilation, and subjective ratings, will help gaining insight into internal motives. Specifically, Sonuga-Barke (2002) predicted that ADHD symptoms are associated with the experience of negative emotions during waiting which in turn leads to more frequent preferences for small immediate rewards. This mediation hypothesis of negative emotions will need to be tested further, also against alternative or complementary hypotheses such as the prediction that delays will be perceived as longer by individuals with ADHD than controls, and that this altered time perception, more than the emotional value of waiting, mediates the link between ADHD symptoms and the relatively strong preference for immediate rewards in individuals with ADHD (e.g., see Barkley, 1997).

Douglas’ early work showed that behavior/performance of individuals with ADHD significantly improves when tasks are made to be more salient, novel, interesting, or stimulating (Douglas, 1985; see also Zentall, 1986; Zentall & Shaw, 1980). While this may suggest that individuals with ADHD may need different contexts or situations than typically developing peers in order to function well and be optimally (intrinsically or autonomously) motivated, an improvement in behavior and performance alone is probably not sufficient to support the conclusion that intrinsic motivation has increased. With the use of novel, interesting, and salient tasks, it could, for example, also be the case that an increase in neurophysiological activation level would explain the improvement in behavior and performance. Therefore, more research that includes interesting tasks complemented with measures of task interest, enjoyment, and intrinsic motivation next to performance measures, may reveal more about motivation qualities in individuals with ADHD.

Additionally, fMRI research on reward anticipation has shown that reductions in ventral striatum activation during the anticipatory phase of the Monetary Incentive Delay (MID) are consistently associated with ADHD symptoms (see for a meta-analysis Plichta & Scheres, 2015). In addition to future longitudinal research needing to address the relevant question *how* this reduced activation is linked to ADHD symptoms (see Gallo & Posner, 2016 for an interesting discussion), it is also as of yet unclear which particular motivational aspects explain this neural finding: do negative emotions related to the delay contribute to this finding? Does reduced motivation to maximize financial gains contribute? Is this reduction in brain activation associated with probability of winning, anticipatory delay duration, or sensitivity to reward magnitude, or to neurophysiologic underactivation in general? Such questions can be clarified in future research by manipulating delay and reward magnitude systematically, for example, and by making use of subjective valence ratings regarding these parameters and physiological measures such as skin conductance. For example, participants who exhibit neural hyposensitivity when anticipating rewards will most likely show weak affective reactions (behavior), less anticipatory brain activation (neural), and will experience less excitement (physiological and as self-reported) compared to participants who have a more positive appraisal of money.

Moreover, in the research of Morsink et al. (in preparation ) it was shown that different measures of task outcome such as performance, self-reported motivation, engagement during free choice paradigm, and task experience are only correlated to a small extent. Therefore, including a combination of the described measurements can elucidate the *Internal motives*, the “missing link” between environment and behavior (see Figure 1). Such measurements could represent measures of intrinsic motivation (including quality of motivation to take part in the research: was the child intrinsically motivated to take part, or did they do it to satisfy their parents’ wish? or both?), task interest and enjoyment, subjective ratings, and physiological and brain measures. Also Luman et al. (2005) discussed the different measurements and the impact thereof: She suggested that explanations for discrepant sensitivity to reinforcement effects between self-reported and observed motivation could include that self-ratings and observations do not tap into the same concept, and that children may have difficulties monitoring their motivation while performing a task. Another possibility that we want to raise here is that observers may have a hard time gauging which internal processes are going on in the participants. In order to capture such internal processes, self-reports appear necessary. However, it needs to be mentioned that self-reports have certain limitations. For example, self-reports are limited to the measurement of conscious motivations, they rely on honest responses from participants and are subject to bias, and they may reflect not only the motivation that triggered an action, but also, for example, conscious feelings that accompany that action. Therefore, it may be best to include a variety of measures of motivation together, such as performance, self-report, and physiological measurements.

## Clinical Implications

A number of specific SDT-based interventions have been developed but not tested in an ADHD population (see for some recent e.g., Aelterman et al., 2013, 2014, 2016; Haakma et al., 2016). Su and Reeve (2011) performed a meta-analysis on 19 studies that examined the effects of intervention programs explicitly focused on autonomy support. This analysis showed that these programs were, overall, effective (effect size 0.63). An illustrative example shows the relevance of basic need satisfaction for individuals with ADHD: Thomassin and Suveg (2012) found that parental autonomy support moderated the relation between high ADHD symptoms and decreased perseverance while solving a puzzle: When parental support was high, the negative association between ADHD symptoms and perseverance was non-significant. However, SDT based interventions have not yet been offered to individuals with ADHD, or at least, such interventions have not yet been documented in scientific publications. We did find unpublished information about SDT being applied to academic achievement in education settings including students with ADHD. For example, Field (2013) presented at the Twelfth Annual Timothy B. and Jane A. Burnett Seminar for Academic Achievement about practical ways in which SDT principles can be applied to support learning in students in general, and in particular to students with ADHD. For example, the cornerstone strategies that she suggested included that teachers and parents become co-learners in collaboration with students, and the use of cooperative learning to enhance the development of positive relationship skills (Parker et al., 2012). She also showed that coaching including instructions that relate to SDT can be effective in students with ADHD (Algozzine et al., 2001; Parker & Boutelle, 2009). Out of 19 studies, 18 found that ADHD coaching improved ADHD symptoms and executive functioning, and 6 reported improved well-being (Ahmann et al., 2018) . More research is needed to examine to what extent SDT elements in coaching are responsible for these effects. Generally, more research on the effects of SDT-interventions is needed. One intervention in particular that would be relevant for children with ADHD is one where teachers are offered an effective autonomy-supportive intervention program (Cheon & Reeve, 2015). Finally, motivational interviewing (MI) has found its way into ADHD interventions for adolescents with ADHD (Sibley et al., 2016, Boyer et al., 2015). Markland et al. (2005) suggest that MI as well as SDT assume that humans have an innate tendency for personal growth. MI provides the supporting factors that stimulate this tendency for growth. The SDT perspective can be used to design research that examines the psychological processes involved in MI (see also Vansteenkiste et al., 2012).

## Conclusion

In sum, we suggest that SDT is a useful framework for the field of ADHD, and new research on motivation can be embedded in this framework. Specifically, we have proposed to extend current research to add *internal motives* including motivation quality and basic need satisfaction as potential key mediators in the relation between environmental factors on the one hand and behavior or symptoms on the other. Also, we have suggested to study potential negative effects of external reinforcers applied in situation of high intrinsic motivation, on outcome measures such as intrinsic motivation, affect, and well-being. Finally, we believe that this framework also carries value for further development of clinical interventions for those with ADHD, such as the SDT-based Autonomy Support Intervention Program. Our hope is that this overview will stimulate future research, so that we will better and more fully understand which role motivation plays in individuals with ADHD, and which circumstances are optimal for individuals with ADHD to feel supported in their basic needs, and to experience autonomous qualities of motivation.
